# Variation in context‐dependent foraging behavior across pollinators

**DOI:** 10.1002/ece3.4303

**Published:** 2018-07-16

**Authors:** Heather M. Briggs, Stuart Graham, Callin M. Switzer, Robin Hopkins

**Affiliations:** ^1^ Department of Organismic and Evolutionary Biology The Arnold Arboretum of Harvard University Boston Massachusetts; ^2^ Centre d'Ecologie Fonctionnelle et Evolutive Montpellier France; ^3^ Present address: Department of Ecology and Evolution University of California Irvine California; ^4^ Present address: Department of Biology University of Washington Seattle Washington

**Keywords:** floral traits, flower color, Lepidoptera, pollinator, pollinator preference

## Abstract

Pollinator foraging behavior has direct consequences for plant reproduction and has been implicated in driving floral trait evolution. Exploring the degree to which pollinators exhibit flexibility in foraging behavior will add to a mechanistic understanding of how pollinators can impose selection on plant traits. Although plants have evolved suites of floral traits to attract pollinators, flower color is a particularly important aspect of the floral display. Some pollinators show strong innate color preference, but many pollinators display flexibility in preference due to learning associations between rewards and color, or due to variable perception of color in different environments or plant communities. This study examines the flexibility in flower color preference of two groups of native butterfly pollinators under natural field conditions. We find that pipevine swallowtails (*Battus philenor*) and skippers (family *Hesperiidae*), the predominate pollinators of the two native Texas *Phlox* species, *Phlox cuspidata* and *Phlox drummondii*, display distinct patterns of color preferences across different contexts. Pipevine swallowtails exhibit highly flexible color preferences and likely utilize other floral traits to make foraging decisions. In contrast, skippers have consistent color preferences and likely use flower color as a primary cue for foraging. As a result of this variation in color preference flexibility, the two pollinator groups impose concordant selection on flower color in some contexts but discordant selection in other contexts. This variability could have profound implications for how flower traits respond to pollinator‐mediated selection. Our findings suggest that studying dynamics of behavior in natural field conditions is important for understanding plant–pollinator interactions.

## 1 INTRODUCTION

Plants solicit pollinator visitation through a variety of floral display traits including color, scent, size, and shape (Schiestl & Johnson, 2013). These traits generally advertise to pollinators the availability of a reward such as pollen and nectar. Flower color is a particularly important trait driving patterns of pollinator visits to plants (Dötterl, Glück, Jürgens, Woodring, & Aas, 2014; Ômura & Honda, 2005; von Frisch, Lindauer, & Daumer, 1914). Extensive studies—both in controlled laboratory settings and natural field conditions—have documented pollinators exhibiting foraging preferences, in which they disproportionately visit flowers of particular colors (Campbell, Bischoff, Lord, & Robertson, 2010; Schemske & Bradshaw, 1999; Thairu & Brunet, 2015; Weiss, 1997). For example, hummingbirds tend to visit red flowers, while moths and bats visit white flowers and bees often prefer yellow or purple flowers. While these associations between pollinator groups and flower color are seemingly ubiquitous, there are numerous exceptions (as described in reference Ollerton et al., 2009). Some of these exceptions are due to pollinators exhibiting flexibility in color preference. Understanding the degree to which pollinators are consistent or flexible in their preference patterns is key to determining the strength and direction of selection pollinators impose on plant populations.

Flexibility in flower color preference can arise through a variety of mechanisms. Bees, flies, and butterflies can alter their innate color preference by learning an association between nectar or pollen reward and a trait such as color (Goulson, Cruise, Sparrow, & Harris, 2007; Gumbert, 2000; Raine & Chittka, 2008; Weiss, 1997). For example, while bumblebees often display an innate preference for blue flowers, they can readily learn to associate a reward with a novel flower color (Gumbert, 2000; Raine & Chittka, 2007). Although rarely studied in the field, these laboratory studies suggest that learning could explain some of the observed variation in flower color preference we see in nature. Plant community characteristics can also contribute to variation in color preference. For example, variation in the degree to which flower color contrasts with the background can drive flexibility in color preference (Osorio & Vorobyev, 2008). In addition, the presence of other morphologically similar flowering species or presence of a dominant pollinator competitor can contribute to context‐dependent flower color preferences (Brosi & Briggs, 2013; Fornoff et al., 2016). Finally, some studies have shown that innate preference and learning of a particular trait, such as color, can vary depending on other aspects of the complex floral display such as scent, size, and shape. For example, strength and direction of preference for a certain flower color can depend on the presence or absence of scent signals (Knauer & Schiestl, 2016; Leonard & Masek, 2014; Russell, Newman, & Papaj, 2016; Yoshida, Itoh, Ômura, Arikawa, & Kinoshita, 2015). For these reasons, it is likely that pollinators in nature display extensive flexibility in floral color preference across plant species and in different communities; and yet, the extent of this flexibility is largely unknown.

The flexibility of pollinator color preference can have important implications for the evolution of floral traits. Pollinator preference leads to increased floral visitation and thus selection for the preferred flower type (Aldridge & Campbell, 2007; Schemske & Bradshaw, 1999). Despite the recognized importance of pollinator preference on plant trait evolution, we are lacking studies that examine the consistency of these pollinator behaviors under field conditions. Furthermore, little attention has been given to how observed preference in one context might vary in other contexts. Understanding flexibility in pollinator behavior can provide insights into the stability of selection on floral traits and the reliability of pollination services across changing environments.

Wild lepidopterans are well suited to investigating flexibility in color preference in a natural field setting because they are important but understudied pollinators (Rader et al., 2016). They exhibit variation in visual systems across families and even species, which could translate to differential selection pressures on flower color (and other traits) within a given community of co‐occurring pollinators and plants (Briscoe, 2008; Stavenga & Arikawa, 2006). In addition, lepidopterans have been shown to display innate color preferences and yet can alter preference through learning (Blackiston, Briscoe, & Weiss, 2011; Kandori, Hirao, Matsunaga, & Kurosaki, 2009). Finally, while largely unexplored, there is some evidence that butterflies can have flexible color preference depending on the environmental context of the display (Kinoshita, Shimada, & Arikawa, 1999). Despite the evidence that butterflies can be flexible in their color preference, very few studies have explored the extent to which they *are* flexible in their color preference in natural systems.

**Figure 1:**
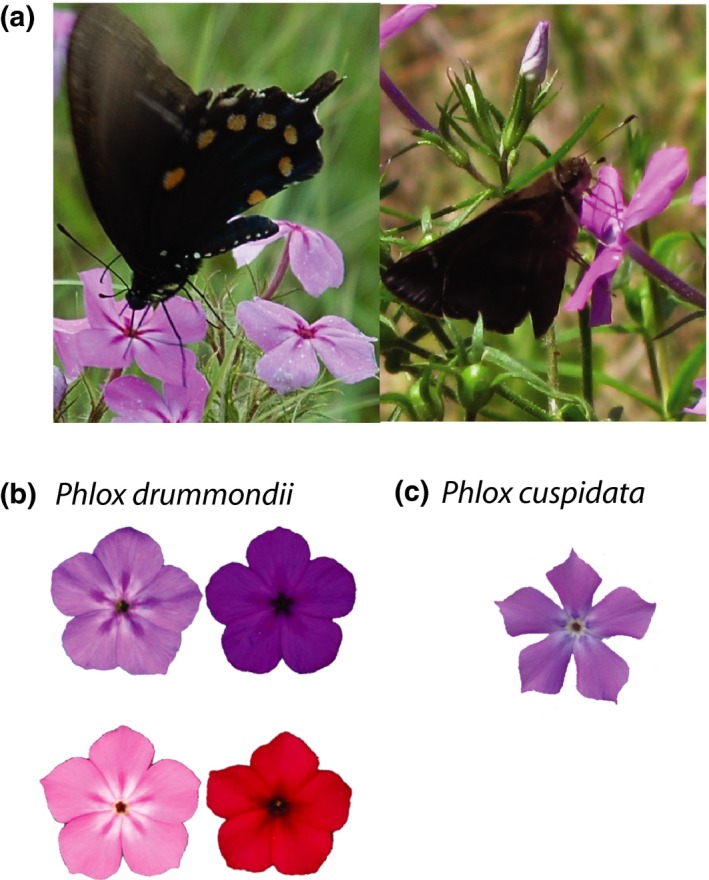
*Phlox drummondii*,* Phlox cuspidata*, and their two predominate pollinators, a pipevine swallowtail and a skipper. (a) Pipevine swallowtail visiting a light‐blue flowered *P. drummondii* flower (left) and a skipper visiting a light‐blue flowered *P. cuspidata* (right). (b) Example four *P. drummondii* morphotypes. Top left, light‐blue; top right, dark‐blue; bottom left, light‐red; bottom right, dark‐red. (c) Example of *P. cuspidata* flower

We investigate the flexibility of flower color preference in two groups of butterfly pollinators that co‐occur in natural communities. In particular, we examine whether color preference changes depending on the plant species present. We observed the foraging behavior of pipevine swallowtails (*Battus philenor*; hereafter pipevine swallowtails, Figure 1a) and a variety of skipper species (family *Hesperiidae*; hereafter skippers, Figure 1a) on experimental arrays composed of combinations of two (naturally co‐occurring) *Phlox* species in a field setting. *Phlox drummondii* (Figure 1b) and *P. cuspidata* (Figure 1c) are the same light‐blue flower color throughout much of their range, but *P. drummondii* has three additional flower color morphologies—light‐red, dark‐red, and dark‐blue (Figure 1b). With this system we can ask how preference for light‐blue flower color changes depending on which plant species is present. We investigate color preference in replicate contrasts between light‐blue and the three other possible *P. drummondii* flower colors, which allows us to decouple flower color from plant species identity and explore the flexibility of color preference in two co‐occurring groups of butterfly pollinators.

In this study, we ask specifically: (a) Is pollinator color preference flexible depending on floral context (i.e., plant species identity or the identity of co‐occurring and morphologically similar plant species)? (b) Do pollinators show similar color preference across different floral contexts?

Our experiment explores flower color preference across the following three distinct floral contexts: (a) within‐species color preference (WS), preference for light‐blue *P. drummondii* compared to *P. drummondii* of the other three colors; (b) between‐species color preference (BS), preference for light‐blue *P. cuspidata* compared to *P. drummondii* of the other three colors; and (c) community context (CC), preference for light‐blue *P. drummondii* compared to the other three *P. drummondii* colors with *P. cuspidata* present in the array.

## 2 MATERIALS AND METHODS

### 2.1 Study system

*Phlox* is a butterfly‐pollinated genus (Levin & Berube, 1972). *P. drummondi* and *P. cuspidata* are annual herbs native to central and eastern Texas that inhabit roadsides, open fields, and pastures. Individuals germinate in late fall or early spring and flower and set fruit from March through June. Both *P. cuspidata* and *P. drummondii* receive up to 95% of their pollination visits from pipevine swallowtails and a variety of skipper species (family *Hesperiidae*; Hopkins & Rausher, 2012, 2014).

*Phlox cuspidata* has light‐blue flowers characteristic of most *Phlox* species. *P. drummondii* also has the same light‐blue flower color across much of its range, but in some eastern and central Texas populations, *P*.* drummondii* has evolved dark‐red, light‐red, and dark‐blue flower colors (Hopkins & Rausher, 2011, 2012) (Figure 1b). Butterfly foraging behavior generates selection on flower color and maintains the flower color polymorphisms across the *P. drummondii* range. In western populations, *P. drummondii* individuals have light‐blue flowers; however, in populations sympatric with the light‐blue‐flowered *P. cuspidata*,* P. drummondii* has evolved dark‐red flowers (Hopkins & Rausher, 2011, 2012, 2014). In the geographic region where light‐blue and dark‐red *P. drummondii* meet, all four flower colors can be found (Hopkins & Rausher, 2014). *P. drummondii* individuals with different flower colors do not differ systematically in other traits [R. Hopkins, unpublished data].

In all years, we collected the plants for this experiment from natural populations throughout the native ranges of *P. drummondii* and *P. cuspidata*. We then grew the plants from seed in glasshouses at the Arnold Arboretum of Harvard University (2015) and at the University of Texas, Austin (2010 and 2012). In all years, we soaked seeds in 500 ppm gibberellic acid for 48 hr to synchronize germination, planted them in water‐saturated Metro‐Mix 360 (Sun Gro Horticulture, Bellevue, WA), and stratified them at 4°C for 7 days. Plants were allowed to germinate and grow in 4 × 4 inch pots in growth chambers set for 14 hr daylight and a 25/22°C day/night temperature regime. We watered and fertilized the plants regularly with Dyna‐Gro Liquid Bloom fertilizer (Dyna‐Gro Nutrition Solutions, Richmond, CA, USA). Each year we transported all plants that were considered to be healthy to The University of Texas Brackenridge Field Laboratory (Austin, TX, USA) for experimentation.

### 2.2 Experimental arrays

**Figure 2:**
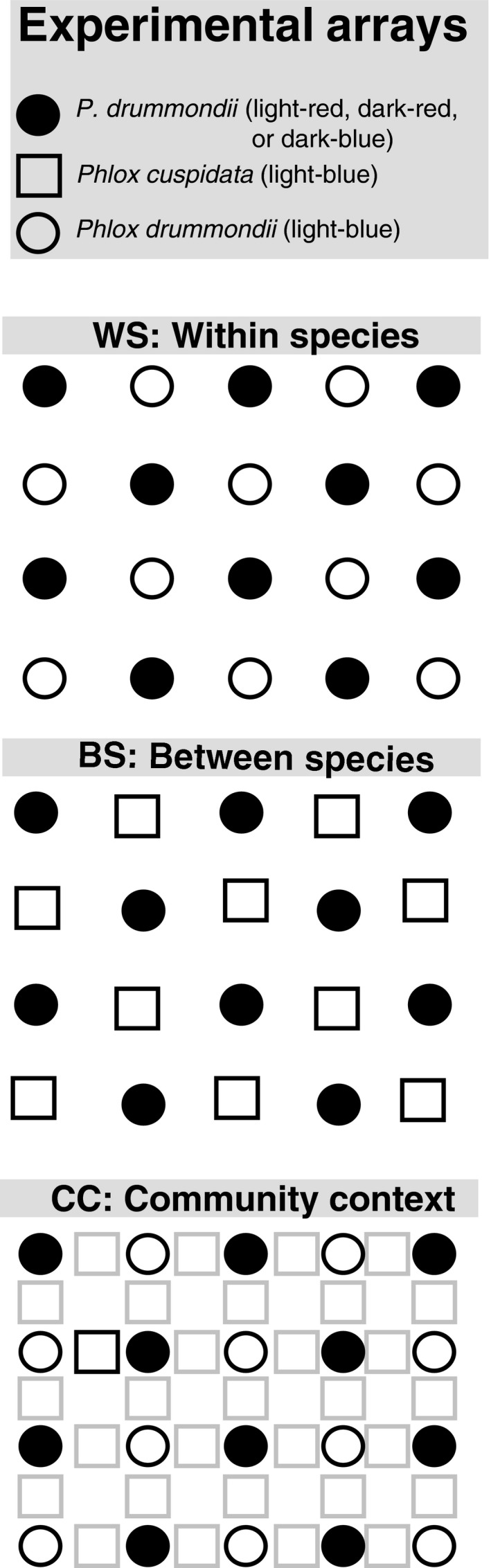
Schematic of the pollinator observation arrays. For each array type, we alternated focal flower colors in a 4 × 5 grid. Within‐species (WS) arrays alternate light‐blue *Phlox drummondii* and *P. drummondii* of one other color (light‐red, dark‐red, or dark‐blue). Between‐species (BS) arrays alternate light‐blue *Phlox cuspidata* and *P. drummondii* of one other color. Community context (CC) arrays alternate light‐blue *P. drummondii* and *P. drummondii* of one other color and include *P. cuspidata* interspersed. Gray boxes (*P. cuspidata*) in CC array indicate that plants were present, but pollinator foraging data were not collected on these plants

During experimentation, we cared for plants in the glasshouse and brought them from the glasshouse into a field at the Brackenridge Field Laboratory between 10 a.m. and 4 p.m. for pollinator observations, and then returned the plants to the glasshouse. We created arrays of potted plants in a 4 × 5 grid, alternating colors (light‐blue and one other *P. drummondii* color), with each pot approximately 20 cm apart. In total, each color had the same number of open flowers (ranging from 518 to 1,254 across days). Due to logistical limitations, we collected foraging data on one floral context per year (see Figure 2 for details). Each experimental array represented one of three floral contexts (described below). Every array contained light‐blue flowers of a focal species and an equal number of *P. drummondii* flowers of one other color (light‐red, dark‐red, or dark‐blue). Across the arrays, we varied both the species identity of the light‐blue flowers and the color of the non‐light‐blue *P. drummondii* flowers in a full factorial design to give a total of nine distinct experimental array types.

Our three floral contexts were as follows: (1) within‐species color preference (hereafter WS), measures color preference for light‐blue *P. drummondii* plants versus other colors of *P. drummondii*; (2) between‐species color preference (hereafter BS), measures color preference for light‐blue *P. cuspidata* plants versus other colors of *P. drummondii*; and (3) community context (hereafter CC), measurescolor preference for light‐blue *P. drummondii* plants versus other colors of *P. drummondii* when *P. cuspidata* is interspersed in the array.

Contexts (1) and (2) differ only in which *Phlox* species has light‐blue flowers. This allows us to test the effect of species identity on light‐blue phenotype preference. Contexts (1) and (3) differ only in the presence/absence of *P. cuspidata*. Foraging visits to *P. cuspidata* were not recorded in this context. This comparison allows us to assess the impact that the presence of a morphologically similar coflowering species can have on pollinator preference.

### 2.3 Pollinator observations

We assessed color preference over 3 years (2010, 2012, and 2015) in the month of May at the Brackenridge Field Laboratory. This site is located in the allopatric range of *P. drummondii*, and wild populations of light‐blue *P. drummondii* exist nearby. The pollinator observations from 2011 and 2012 are included in previous publications investigating selection on *Phlox* (Hopkins & Rausher, 2012, 2014).

We recorded the foraging behavior of free‐flying butterflies on arrays of live *Phlox* plants to examine color preference in varying contexts. We had a single observer record pollinator observations from 10 a.m. and 4 p.m. on at least 2 days per array. We only recorded foraging of a single pollinator at a time on an array. Each pollinator was identified as pipevine swallowtail, skipper, or “other.” While many skipper species have been recorded at the Brackenridge Field Laboratory, we believe, based on visual recognition, that only five of those species (*Thorybes pylades*,* Erynnis horatius*,* Copaeodes aurantiaca*,* Atalopedes campestris*, and *Hylephila phyleas*) likely visited our arrays. Skipper butterflies are difficult to identify on the wing, and all five species are of similar shape and size. For each pollinator, we recorded the color and species of each flower visited. Pollinator visits were counted only if the pollinator's proboscis was seen entering a corolla. From these data, we calculated the total number of plants visited of each color by each pollinator.

### 2.4 Data analysis

Only plant visits from pipevine swallowtails and skippers were included in our analyses. Other pollinator species (~5% of total visits) were excluded because of their small sample sizes and because their behavior on flowers suggested that they could not access the pollen or nectar rewards of the flowers. Because arrays contained equal numbers of each compared color, pollinator preference could be measured as the proportion of total floral visits to the light‐blue focal species. A value of 0.5 indicates no preference, and a value greater than 0.5 indicates preference for light‐blue flowers.

**Figure 3:**
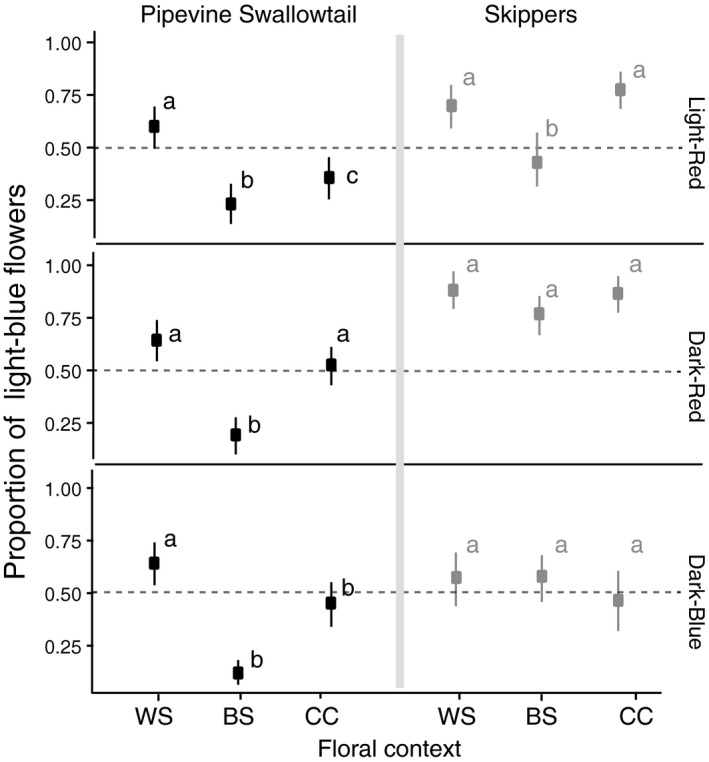
Context‐dependent flower color preferences vary by pollinator species: Mean proportion of visits to light‐blue flowers versus other color flowers across the three contexts (see Figure 2 for full description of floral contexts) for pipevine swallowtails (in black) and skipper butterflies (in gray). 95% bootstrap CIs are plotted around the mean. Letters indicate significant differences in color preferences between the three contexts for each butterfly group and color type. Model results from the contrasts comparing the preference across contexts are displayed in Table 1

**Figure 4:**
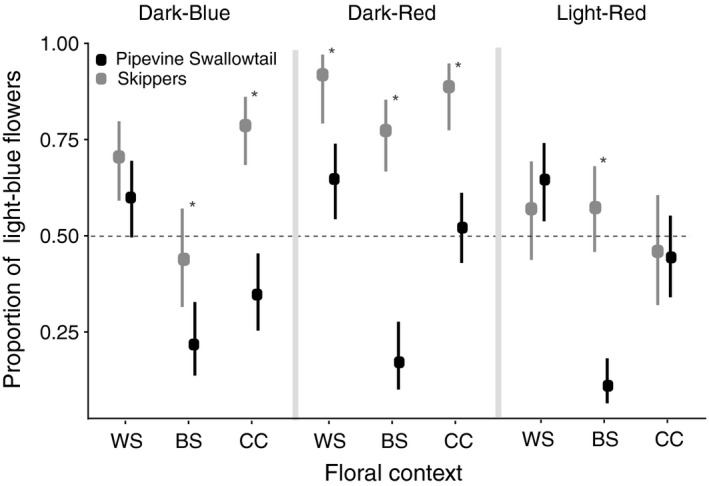
Butterfly groups differ in flower color preference across floral contexts. Mean proportion of visits to light‐blue flowers versus other color flowers across the three contexts (see Figure 2 for full description of contexts) for pipevine swallowtails (in black) and skipper butterflies (in gray). 95% bootstrap CIs are plotted around the mean. Asterisks indicate significant differences in color preference between the two pollinator groups within a given context. Model results from the contrasts comparing the two butterfly groups are displayed in Table 2

We used GLMMs with binomial errors and a logit link function in the lme4 R package to model the number of visits a pollinator makes to light‐blue flowers versus other color flowers (Bates, Maechler, & Bolker, 2012). We included three fixed effects in our model: floral context (WS, BS, CC; see Figure 2), pollinator type (pipevine swallowtail or skipper), and other *P. drummondii* flower color (light‐red, dark‐red, dark‐blue; see Figure 1b). We included both pollinator individual and date of data collection as random effects in the model. The date of data collection was included because samples taken on the same array type on different days could not be considered independent. We assessed the flexibility of pollinator color preference with a model including all two‐way interactions and the three‐way interaction between our three fixed effects. We determined that a model including the three‐way interaction of the three main effects was the best‐fit model through a likelihood ratio test. To understand the specific foraging context by color‐type‐by‐pollinator‐type interactions causing this significant three‐way interaction, we ran pairwise post hoc tests using the glht() function in the multcomp R package (Hothorn, Bretz, Westfall, Heiberger, & Schuetzenmeister, 2013). We did not adjust for multiple tests because our contrasts were targeted to test specific a priori hypotheses (Benjamini, 2010). Implementing a correction does not change the interpretation of our results. Our contrasts were targeted to assess (a) significant differences in flower color preference within a given color type across all three foraging contexts for a given pollinator; and (b) differences in color preference between the two pollinator types in a particular foraging context (see Tables 1 and 2 for pairwise comparisons). Due to the complicated nature of displaying significant results from a three‐way interaction, we display the data in two separate figures (Figures 3 and 4).

**Table 1:** Results from post hoc pairwise comparisons testing how color preference changes across species contexts within each pollinator group using generalized linear mixed‐effects models with binomial errors

		Context comparisons	Estimate	*SE*	*T*_Stat_	*p*
Pipevine swallowtail	Dark‐blue	BS–WS	−1.684	0.355	−4.738	**<0.001**
		CC–BS	0.649	0.374	1.738	0.082
		CC–WS	−1.034	0.319	−3.241	**0.001**
	Dark‐red	BS–WS	−2.185	0.385	−5.672	**<0.001**
		CC–BS	1.661	0.365	4.546	**<0.001**
		CC–WS	−0.523	0.292	−1.791	0.073
	Light‐red	BS–WS	−2.690	0.377	−7.137	**<0.001**
		CC–BS	1.863	0.372	5.009	**<0.001**
		CC–WS	−0.827	0.319	−2.59	**0.010**
Skippers	Dark‐blue	BS–WS	−1.117	0.372	−3.003	**0.003**
		CC–BS	1.544	0.382	4.045	**<0.001**
		CC–WS	0.428	0.369	1.159	0.247
	Dark‐red	BS–WS	−1.193	0.617	−1.933	0.053
		CC–BS	0.837	0.504	1.66	0.097
		CC–WS	−0.356	0.697	−0.511	0.610
	Light‐red	BS–WS	0.013	0.360	0.035	0.972
		CC–BS	−0.457	0.383	−1.193	0.233
		CC–WS	−0.444	0.406	−1.094	0.274

*Notes*

See Figure 3 for details on which color was preferred in each context.

Bolded text in columns indicate significant differences in color preferences between contexts for a given pollinator group.

**Table 2:** Results from post hoc pairwise comparisons testing whether two pollinator groups differ in their color preference across foraging contexts using generalized linear mixed‐effects models with binomial errors

Color	Context	Estimate	*SE*	*T*_Stat_	*p*
Pollinator comparison pipevine swallowtail–skippers					
Dark‐blue	BS	−1.035	0.338	−3.064	**0.002**
	WS	−0.468	0.259	−1.806	0.071
	CC	−1.93	0.335	−5.764	**<0.001**
Dark‐red	BS	−2.805	0.384	−7.302	**<0.001**
	WS	−1.813	0.564	−3.214	**0.001**
	CC	−1.98	0.437	−4.528	**<0.001**
Light‐red	BS	−2.384	0.327	−7.292	**<0.001**
	WS	0.319	0.308	1.037	0.300
	CC	−0.064	0.305	−0.209	0.834

*Notes*

See Figure 4 for more details about which color was preferred in each context.

Bolded text indicates significant differences in color preferences between swallowtail and skippers within a given context.

## 3 RESULTS

Over 3 years, we observed a total of 2,441 visits from 312 pipevine swallowtail butterflies and 962 visits from 308 skipper butterflies foraging on our experimental arrays (Supporting Information Table S1). The model that best predicted color preference of the two butterflies was the full model that included a three‐way interaction between the fixed effects (foraging context, pollinator type, and flower color type). This model revealed a highly significant three‐way interaction (see Tables 1 and 2 for pairwise comparisons). For the purpose of this study, we were interested in understanding how color preference and floral context interact to shape flexibility in pollinator color preference. Furthermore, we wanted to know whether the two main pollinators of *Phlox* show different or similar color preference within each foraging context. As such, we report post hoc tests relevant for answering those specific questions below.

### 3.1 Context‐dependent preference

First, we were interested in determining whether pollinator color preference is flexible depending on floral context (i.e., plant species identity or the identity of co‐occurring and morphologically similar plant species). Pipevine swallowtails showed significant flexibility in color preference depending on floral context, and skippers showed little to no flexibility of color preference across the different floral contexts in our study (Figure 3). For pipevine swallowtails, the strength and direction of preference are significantly different between floral contexts. This flexibility is evident in each of the three flower color comparisons (Figure 3, Table 1). For example, in the light‐red arrays, pipevine swallowtails have a preference for light‐blue flowers when the two flower colors are the same species (WS) but preference for light‐red color when the two flower colors are different species (BS). Furthermore, swallowtails do not exhibit a color preference when the morphologically similar *P. cuspidata* is present in the array (CC). We observed qualitatively similar patterns of color preference flexibility for the dark‐red and dark‐blue color arrays as well (see Figure 3 and Table 1 for contrast results).

In contrast, skippers do not exhibit significant flexibility in color preference and are generally consistent with color preference regardless of the floral context (Figure 3, Table 1). When choosing between light‐red and light‐blue, skippers display no color preference regardless of the *Phlox* species present contexts. Skippers exhibit a strong preference for light‐blue flowers over dark‐red flowers in all three floral contexts, regardless of the flower species identity. Skippers in the dark‐blue arrays exhibit preference for light‐blue flowers in two of the three contexts and weak to no preference when light‐blue *P. cuspidata* is paired with dark‐blue *P. drummondii* (SI).

### 3.2 Pollinator contrasts

Second, we were interested in determining whether the two primary pollinators of *Phlox* show similar color preference across different floral contexts. Floral context dictates whether or not the pipevine swallowtail and skippers show similar color preference (Figure 4, Table 2). In the within‐species (WS) contrasts, skippers and pipevine swallowtails show similarly strong preference for light‐blue *P. drummondii* flowers compared to the other colors of *P. drummondii*. However, when the morphologically similar *P. cuspidata* is paired with *P. drummondii* in the between‐species contrasts (BS), the two butterflies show significantly different color preferences for all three color‐type arrays. In the same way, when the morphologically similar *P. cuspidata* is present in both the dark‐blue and dark‐red color arrays, the two pollinators have significantly different color preference for light‐blue *P. drummondii* flowers. These findings suggest that while swallowtails appear to exhibit color preference based on species identity, preferring all colors of *P. drummondii* over the light‐blue *P. cuspidata* flowers, skippers appear to rely heavily on flower color to guide foraging preferences.

## 4 DISCUSSION

We found that two groups of generalist pollinators, pipevine swallowtails and skippers, vary in the consistency of their color preference while foraging in a natural field experiment. This variation in flexibility across the pollinators means that pollinator‐driven selection on flower color is inconsistent across floral contexts. Our results are based on observing wild butterflies, which have unknown foraging experience, foraging on arrays of native plants in their natural habitat. This experiment was performed using two *Phlox* wildflower species that depend on these pollinators for as much as 95% of their pollination visits, suggesting this behavioral variability has important implications for plant evolution.

Pipevine swallowtails showed context‐dependent color preference such that the strength and direction of their color preference depend on both the species identities of plants that differ in color and the presence or absence of another, morphologically similar, plant species in the area. For example, we found that these butterflies show preference for light‐blue over dark‐red under one floral context, no preference in another context, and preference for dark‐red over light‐blue in the third context. We found similar inconsistencies in the direction and strength of preference when pipevine swallowtails choose between light‐blue and light‐red as well as light‐blue and dark‐blue flowers. These results suggest that pipevine swallowtails could use other traits and environmental signals in addition to color to make foraging decisions and are therefore flexible in their color preference under naturally variable conditions.

In contrast, the skipper butterflies did not show context‐dependent color preference. For all three color comparisons, we found that skippers displayed similar strength and direction of color preference regardless of the species being compared or whether a morphologically similar species was present in the array. This strong preference consistency suggests that skippers use color as an important foraging cue and are relatively inflexible in their preference for particular colors.

Previous studies in this system demonstrated that flower color variation across the range of *P. drummondii* is maintained by pollinator‐mediated selection (Hopkins & Rausher, 2012, 2014). Dark‐red flower color is favored in populations sympatric with *P. cuspidata* because pollinator behavior decreases costly hybridization between the two species when they have different flower colors (Hopkins & Rausher, 2012). In addition, pollinator behavior in allopatry favors light‐blue flower color and thus maintains the ancestral phenotype in western *P. drummondii* populations (Hopkins & Rausher, 2014). In this system, understanding the flexibility of pollinator behavior across plant species and community contexts is important to determine the stability of selection on flower color across geographic space and time. Our study suggests that much research is needed to understand whether flexible color preference in pipevine swallowtails leads to spatially or temporally varying selection on flower color. For example, do pipevine swallowtails in the sympatric range actually discriminate against light‐blue *P. drummondii* plants because *P. cuspidata* is in the community? While *P. drummondii* and *P. cuspidata* have similar light‐blue flowers, the flowers differ both in size (*P. cuspidata* has smaller flowers) and nectar amount (*P. cuspidata* has lower nectar volume and sugar concentration compared to *P. drummondii*) (R. Hopkins, unpublished data). Variation in traits other than flower color could lead to context‐dependent preferences in pipevine swallowtails. This would suggest an additional mechanism through which pollinator‐mediated selection acts on flower color.

Much of what we have learned about color preference in butterflies comes from laboratory studies, often explored through the use of artificial flowers (Kelber & Pfaff, 1997; Kinoshita et al., 1999; Weiss, 1997; Weiss & Papaj, 2003). Therefore, despite the wealth of information we have about butterfly color preference, we know little about how these behaviors translate to natural systems. From these laboratory studies, it is evident that many butterflies display innate color preferences as well as learned associations between colors and nectar rewards. For example, pipevine swallowtails have an innate preference for blue flowers over yellow in the laboratory (Weiss, 1997). In our study, we found that while pipevine swallowtails preferred blue flowers of one species (*P. drummondii*), they were strongly deterred by the blue flowers of another co‐occurring species (*P. cuspidata*) and ultimately displayed preference for blue flowers based on species identity. These results suggest that our understanding of pipevine swallowtail flower color preference from the laboratory does not necessarily translate to behavior we observed in the field and that these butterflies are likely using other cues in addition to color (such as shape or scent) to guide their foraging preferences. In contrast, we found that skippers more consistently base their foraging decisions on flower color, as color preference did not appear to be influenced by floral context. The indiscriminate color‐based preference that skippers exhibit could have important implications for co‐occurring plants in the community. Skippers may be more likely than swallowtails to move between plant species with the same flower color, transferring heterospecific pollen in the process. The few field studies that examine butterfly color preference suggest that context‐dependent color preference may be common (Clements, 1923; Pohl, Van Wyk, & Campbell, 2011) making the case for future studies that explore color preference in natural contexts.

Pollinator preference for particular floral traits exerts selective pressures on plants. It is therefore of primary interest to understand the extent to which co‐occurring pollinators exert either similar or disparate selective forces on plants. In this study, we found that in some foraging contexts, pipevine swallowtails and skippers exhibit the same color preferences, and in other contexts, we found that the two pollinators display disparate color preferences. In other words, whether or not the two pollinator groups impose concordant selection on flower color depends on the plant species being compared and the background community of plant species. In specialized plant–pollinator interactions, conclusions as to how a pollinator acts as an agent of selection on specific floral traits can be relatively straightforward (i.e., Muchhala & Thomson, 2009). However, most plants are visited by multiple pollinator species and the strength and direction of selection on multiple pollinators impose on floral traits are rarely assessed. Furthermore, the composition of the pollinator visitors can vary both spatially (Gomez, Abdelaziz, Lorite, Jesús Muñoz‐Pajares, & Perfectti, 2010) and temporally (CaraDonna et al., 2017), further complicating our understanding of how pollinator variation drives patterns of selection on floral traits. Understanding the flexibility of pollinator behavior across plant species and community contexts is crucial for determining the dynamics of selection on flower color across geographic space and time.

The two butterfly groups in our study showed different degrees of flexibility in their color preference. This variation in color preference can be due to a number of factors including differences in visual systems and/or differential learning abilities. Unlike most other groups of pollinators, visual pigments of butterfly eyes vary across families and even species. This means that butterfly individuals of different species can both collect and perceive spectral information in different ways. It is not surprising that this variation in color perception can lead to differences in innate color preferences, the ability to learn new colors associated with rewards, and the degree to which color preference will be context‐dependent and influenced by the environment (Blackiston et al., 2011; Briscoe, 2008). While some butterflies have red visual receptors, it appears that skippers do not, likely leading to passive discrimination against red colored flowers (Briscoe & Chittka, 2001). At present, there are no studies examining the pipevine swallowtail visual system, but closely related species exhibit exceptional long‐wavelength visual abilities (Arikawa, 2003; Takemura, Kinoshita, & Arikawa, 2005). Future studies that link flexibility in preference to variation in visual systems will add an invaluable mechanistic understanding of how butterflies can impact selection on plant traits.

Conducting behavioral trials in natural systems is complex and challenging and as with most studies, our design involved some trade‐offs. Because this study was part of a larger, project aimed at characterizing pollinator‐mediated selection, we collected foraging data on one floral context per year. While this design could lead to differences in abiotic conditions, with the potential to impact the number and species composition of floral visitors, we saw a comparable number of each butterfly group across each year and throughout the sampling period Supplemental Table 1. Furthermore, we expect that if there was a “year” effect in our experiment, we would be unlikely to see such strong and consistent patterns within the two butterfly groups. This study clearly does not represent the breadth of possible plant contexts that a pollinator might encounter in the wild. Rather, it highlights the potential for two co‐occurring butterfly groups to vary in their behavioral flexibility, with interesting implications for plant trait evolution. In addition, because we chose not to destructively sample the pollinators visiting our arrays, we were not able to identify the skippers in this study to species. As such, we are not able to rule out the possibility that there could be species‐specific preferences that were masked by our data pooling. Finally, other pollinator behaviors such as constancy are important for plant trait evolution. While we acknowledge that constancy is very important, for simplicity we decided to focus on color preference for this study. Future studies that include constancy will undoubtedly add important insight into how flexible foraging behavior impacts plant trait evolution in the field.

The frequency and pattern of pollinator foraging, as well as the composition of both pollinator and plant communities, can have a direct impact on plant reproductive success and the evolution of plant traits. Many laboratory studies suggest that pollinators display innate and learned color preferences and that these preferences can be flexible, but our study is one of few that explores the flexibility of color preference in the field. Therefore, it remains unclear how results from laboratory studies translate to behavior in natural systems. Our study reveals that two butterfly groups that provide the majority of pollination visitation to two native wildflowers display different flexibility in color preference and, in the case of the pipevine swallowtail, behave in ways that might be difficult to predict from laboratory studies. This study enhances our understanding of whether and how pollinators display flexible foraging preferences in the wild. Future studies that combine descriptions of visual systems with critical behavioral assays in the laboratory and in natural environments will allow us to understand the prevalence and mechanisms underlying flexibility in pollinator foraging behavior.

## CONFLICT OF INTEREST

None declared.

## AUTHOR CONTRIBUTIONS

RH and SG designed the study. RH, SG, and HB collected the field data. HB, CS, and RH analyzed and interpreted data. HB and RH wrote the manuscript.

## DATA DEPOSITION

Data available from the Dryad Digital Repository: https://doi.org/https://doi.org/10.5061/dryad.12t657f.
