# Supplementary material for: Variation in context‐dependent foraging behavior across pollinators
Source: Ecol Evol. 2018 Jul 16;8(16):7964–73. doi: 10.1002/ece3.4303 (PMC6144987; doi:10.1002/ece3.4303)
Supplement: Supplementary file 1 [file ECE3-8-7964-s001.docx]

**Supplemental Table 1:** Number of butterfly individuals recorded in each array and floral context.

|  | **Floral Context** | **Number of Swallowtail individuals** | **Total visits to LB** | **Total visits to other color** | **Number of Skipper individuals** | **Total visits to LB** | **Total visits to other color** |
| --- | --- | --- | --- | --- | --- | --- | --- |
|  | **WS (2015)** |  |  |  |  |  |  |
| **Color** | Dark Blue (N=3) | 53 | 159 | 122 | 63 | 89 | 42 |
|  | Dark Red (N=3) | 37 | 139 | 80 | 30 | 40 | 4 |
|  | Light Red (N=3) | 38 | 124 | 74 | 41 | 52 | 39 |
|  |  |  |  |  |  |  |  |
|  | **BS (2012)** |  |  |  |  |  |  |
| **Color** | Dark Blue (N=3) | 26 | 34 | 157 | 22 | 59 | 66 |
|  | Dark Red (N=4) | 19 | 19 | 92 | 40 | 91 | 27 |
|  | Light Red (N=3) | 31 | 22 | 159 | 50 | 88 | 61 |
|  |  |  |  |  |  |  |  |
|  | **CC (2011)** |  |  |  |  |  |  |
| **Color** | Dark Blue (N=4) | 37 | 117 | 191 | 30 | 105 | 35 |
|  | Dark Red (N=3) | 41 | 300 | 266 | 17 | 58 | 9 |
|  | Light Red (N=2) | 30 | 174 | 212 | 15 | 47 | 50 |
|  |  |  |  |  |  |  |  |
